# Frequency and Knowledge of Analgesics Self-Use and Their Adverse Effects in the Eastern Province of Saudi Arabia

**DOI:** 10.7759/cureus.33344

**Published:** 2023-01-04

**Authors:** Baqer A Almohammed

**Affiliations:** 1 Medicine, Al-Jabr Eye and Ear, Nose, Throat (ENT) Hospital, Al-Ahsa, SAU

**Keywords:** pharmacology, self-use, over the counter, saudi arabia, non-steroidal anti-inflammatory drugs

## Abstract

Background

Paracetamol and non-steroidal anti-inflammatory drugs (NSAIDs) are among the most commonly used over-the-counter (OTC) medications, both locally in Saudi Arabia (SA) and globally. They are widely available and can be easily obtained; however, the potential health risks of these drugs are well-documented. This study aimed to measure the frequency of analgesics’ self-use and assess the general population’s knowledge of their adverse effects.

Methodology

This is a descriptive, cross-sectional study that was conducted through an online self-administered questionnaire. It targeted adults who are non-healthcare professionals living in the eastern province of SA.

Results

The sample consisted of 345 participants, of which 196 (56.8%) were male and 149 (43.2%) were female. The most self-used medication was paracetamol at 91%, followed by ibuprofen at 38.8%. Although the prevalence of self-use was high, a low frequency of repeated use was evident, as 49.3% of the participants rarely used them and 19.4% used them only every few months. There was a significant association between the female gender, lower levels of education, and a higher frequency of repeated use of analgesics. About 54.5% of the participants recognized three side effects or fewer, while 90 (26.1%) of them showed knowledge about four to six side effects.

Conclusions

Considering that paracetamol and NSAIDs are easily procurable OTC, the knowledge of the general population about their harmful adverse effects needs to be enhanced, specifically that of the most vulnerable patient groups.

## Introduction

Paracetamol and non-steroidal anti-inflammatory drugs (NSAIDs) are used to treat various pathological conditions due to their analgesic, antipyretic, and anti-inflammatory effects [1]. These therapeutic effects are achieved mainly through the inhibition of the cyclooxygenase enzyme, which is responsible for prostaglandins biosynthesis [2].

Based on the reported statistics, NSAIDs are widely used around the world, with around 30 million individuals utilizing them on a daily basis globally [1]. Similarly, their use is also prevalent locally in Saudi Arabia (SA), as analgesics constitute 67% of the top ten used drugs in the region, specifically, diclofenac [3]. Furthermore, analgesics and antipyretics account for 41.8% of over-the-counter (OTC)-obtained medications in Riyadh, SA [4]. Acetaminophen, ibuprofen, and diclofenac are the most self-used medications by Saudis with cardiovascular diseases (CVDs) [5].

Although they are commonly prescribed as an adjunct and/or first-line drug for various pathologic conditions, they should be prescribed or ingested (as an OTC drug) with caution, as courses of just a few days, even at doses within the prescribing recommendations, can be associated with serious adverse effects in susceptible patients [6-8]. More specifically, NSAIDs are strongly associated with gastrointestinal (GI) toxicity [6]. NSAID users are at three times greater risk of developing gastric ulcers, GI bleeding, and, in some advanced cases, gastric perforation [6,7]. Moreover, NSAIDs can induce several forms of renal failure in 1-5% of users [8]. This includes the acute deterioration of renal function, renal papillary necrosis, or acute interstitial nephritis [8]. Chronic NSAID abuse makes one more prone to chronic nephritis [8]. In addition, it was also proven that chronic NSAID abuse increases the likelihood of the development of certain cardiovascular adverse effects such as hypertension, myocardial infarction, and heart failure [9]. Hence, the American Heart Association advises users to avoid long-term NSAID self-use without consultation with a healthcare professional [9].

Over 100,000 patients are hospitalized every year in the United States due to NSAID-induced severe GI adverse effects; of these, 15% die of these drug-induced conditions [10]. A study in Germany of patients who were hospitalized due to drug-induced conditions demonstrated that most of the self-medication-related adverse effects were GI adverse events induced by NSAIDs [11]. However, although paracetamol is relatively considered safer than NSAIDs, its abuse and overdose can lead to hepatotoxicity and even liver failure [12].

According to numerous studies, chronic NSAID self-use increases the risk of developing serious adverse effects [6-11]. Given the lack of information among the general population, understanding the attitude and perception of the population in the eastern province of SA toward the potential adverse effects of NSAID self-use is certainly a matter of concern. This concern is due to the potential negative impact of NSAID self-use on the health and quality of life of many patients, which, consequently, leads to an increased medical burden on healthcare systems and a higher economical burden on hospitals. In this regard, this study aimed to measure the frequency of paracetamol and NSAID self-use among the general population of the eastern province of SA, as well as their knowledge of their potential side effects.

## Materials and methods

Study design

This is a descriptive, cross-sectional, questionnaire-based study. It was conducted with the general population of the eastern province of SA. The survey was prepared using the most relevant and previously validated surveys from the literature, although with a few modifications [13,14]. The study procedure followed the Declaration of Helsinki guidelines.

Setting

The study was conducted in the eastern province of SA through social media platforms. Eligible participants who fulfilled the inclusion and exclusion criteria were invited to participate in the study. The surveys were divided between males and females and between city and village residents.

Study population

Saudi adults in the eastern province of SA who have used paracetamol and NSAIDs in their lifetime were recruited for the study. The inclusion criteria included participants who were aged 18 or older and non-healthcare professionals. Participants aged 18 or less, who were healthcare professionals, and of non-Saudi nationality (due to the language barrier) were excluded.

Sampling

A minimum sample size of 342 was required. This value was calculated using Epi‑Info™ software, version 7.2. This calculation was based on a 5% marginal error and 95% confidence interval and estimated 33.33% proportion of users who have knowledge about paracetamol and NSAIDs’ adverse effects, drawing from a previous similar study [13]. A convenience sampling technique was adopted to choose eligible participants.

Study procedure

After obtaining the participants’ consent to utilize their data in the research, they were invited to fill out an online self-administered questionnaire. The questionnaire was designed to address three main categories: the first category is sociodemographic data, which included information on the age, gender, marital status, residency, educational level, and employment status of the participants; next, the participants answered questions related to the frequency of analgesics self-use, type of the drug used, the reason for use of this drug, and the source from which they came to know about these drugs. Finally, the participant’s knowledge of the side effects and contraindicated conditions of the most common NSAIDs and paracetamol was evaluated using an 11-item questionnaire.

A pilot study was conducted with 20 participants to check the reliability of the questionnaire. The questionnaire scored 0.745 on Cronbach’s alpha reliability analysis, indicating acceptable reliability. This study was conducted from October to November 2022.

The primary outcome intended was to assess if self-users of analgesics have basic knowledge about their adverse effects and contraindications. The secondary outcomes included measuring the frequency of analgesic self-use and determining the correlation between sociodemographic factors and high self-use frequency and the level of knowledge.

Data analysis and management

All the data were collected as a softcopy. The participants’ confidentiality was preserved as no personal identifiable information of any participant was recorded. The entire statistical analysis was carried out using the Statistical Package for the Social Sciences (SPSS) software, version 19. Frequencies, proportions, and a Chi-square test were utilized to present and compare the qualitative variables. A P-value less than 0.05 was considered significant.

## Results

The total number of participants in the study sample was 345. Table 1 demonstrates the sociodemographic characteristics of the participants. The 36-45 age group was the largest with 78 (22.6%) participants, while the 56 or older age group was the smallest with 54 (15.7%) participants. In terms of gender distribution, 196 (56.8%) were male and 149 (43.2%) were female. Most participants were married, specifically 267 (77.4%). Regarding the place of residence, 169 (49%) were city residents and 176 (51%) were village residents. Most of the study participants were distributed between university degree holders at 209 (60.6%) participants and high school graduates at 105 (30.4%) participants. There was a significant association between a good level of knowledge and being a city resident (p = 0.030).

**Table 1:** Sociodemographic characteristics of the participants (n = 345). *P-value < 0.05 is significant.

Variable	Frequency	Proportion (%)	Level of knowledge		P-value (level of knowledge)	P-value (self-use frequency)
			Good	Poor		
Age						
18-25 years	69	20	32	37	0.731	0.359
26-35 years	73	21.2	33	40		
36-45 years	78	22.6	29	49		
46-55 years	71	20.6	32	39		
56 years or older	54	15.7	21	33		
Gender						
Male	196	56.8	82	114	0.739	0.017*
Female	149	43.2	65	84		
Marital status						
Married	267	77.4	110	157	0.327	0.244
Not married	78	22.6	37	41		
Residence						
City	169	49	82	87	0.030*	0.463
Village	176	51	65	111		
Level of education						
Elementary school	4	1.2	0	4	0.537	0.000*
Intermediate school	23	6.7	10	13		
High school	105	30.4	46	59		
University degrees	209	60.6	89	120		
No formal education	4	1.2	2	2		
Employment status						
Employed	168	48.7	70	98	0.730	0.688
Not employed	177	51.3	77	100	0.730	0.688

Table 2 shows the OTC analgesics most frequently used by the study population. The most used medication was paracetamol at 91% (314), followed by ibuprofen at 38.8% (134). The least used medication was naproxen, with only 2.6% (2) of the study population reporting its usage.

**Table 2:** Most self-used analgesics (multiple choice).

Medication	Frequency	Proportion (%)
Paracetamol	314	91
Ibuprofen	134	38.8
Diclofenac	57	16.5
Aspirin	28	8.1
Naproxen	2	0.6
Celecoxib	9	2.6
Did not use any without a prescription	24	7

**Figure 1:**
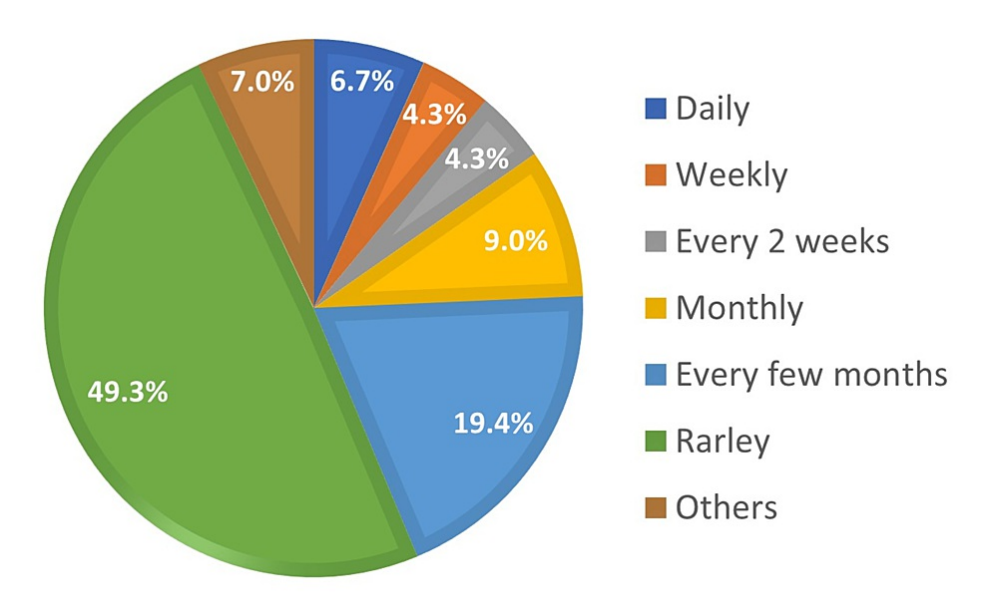
Frequency of analgesics self-use (n=345).

Figure 1 illustrates the frequency of analgesic self-use by the participants. The lower frequency rates were the most reported: 49.3% (170) rarely used these analgesics and 19.4% (67) used them only every few months. On the other hand, only 6.7% (23) reported daily usage. There was a significant association between a high frequency of self-use and the female gender (p = 0.017) and between a low frequency of self-use and higher levels of education (p = 0.000) (Table 1).

**Figure 2:**
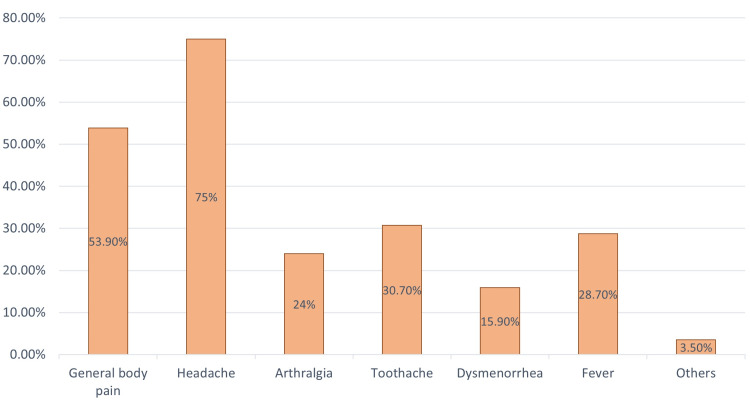
The reasons behind analgesics self-use (multiple choice).

**Figure 3:**
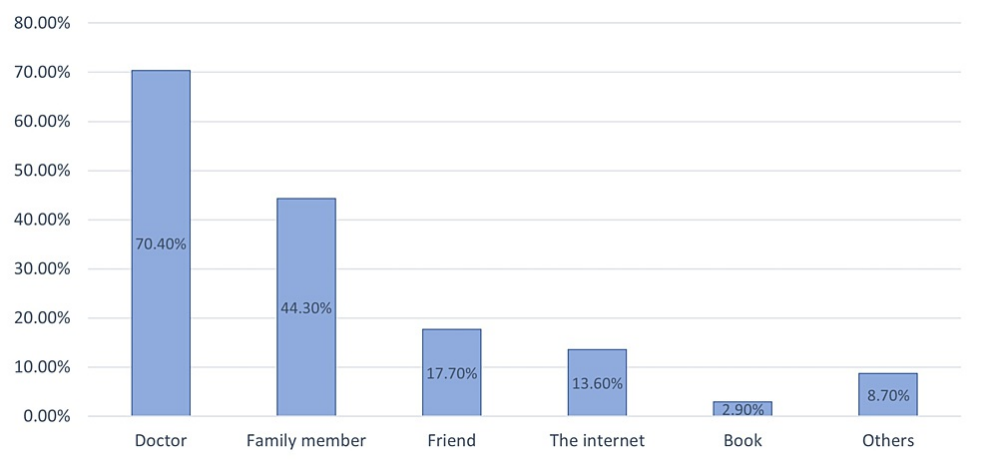
Sources of first knowledge about analgesics (multiple choice).

Figure 2 illustrates the most common reasons behind the OTC use of analgesics. The three leading causes were headache (259, 75.1%), followed by general body pain (186, 53.9%), and toothache (106, 30.7%). Figure 3 is an illustration of the sources from which the participants came to know about these drugs. A doctor was the most prevalent source for 243 (70.4%) participants, followed by a family member for 153 (44.3%) participants.

Table 3 and Table 4 highlight the detailed results of participants’ answers to the questions that were assessing their knowledge regarding the side effects and contraindications of NSAIDs and paracetamol. “NSAIDs’ long-term use may damage the kidneys.” was the most correctly identified side effect, with 259 participants (75.1%) recognizing this side effect. However, 67 (19.4%) did not know any of the side effects; 188 (54.5%) recognized only three side effects or fewer; and 90 (26.1%) recognized about four to six side effects. Regarding the relative contraindications to NSAID self-use, 63.5% (219) recognized that poor kidney function is a contraindication, while 56.6% (195) knew that a person with a gastric ulcer should avoid frequent NSAID usage, which was the second-highest correctly identified contraindication. In contrast, only 14.2% (49) agreed that bronchial asthma is a possible contraindication to frequent NSAID intake, which was the least recognized contraindication.

**Table 3:** Assessment of general population’s knowledge about the possible adverse effects of NSAIDs and paracetamol.

Statement	True	False	I do not know
NSAIDs can cause gastric or intestinal bleeding.	131 (38%)	63 (18%)	151 (43.8%)
NSAIDs can cause skin rashes.	66 (19.1%)	84 (24.3%)	195 (56.5%)
NSAIDs’ long-term use may damage the kidneys.	259 (75.1%)	9 (2.6%)	77 (22.3%)
NSAIDs’ long-term use may raise blood pressure.	93 (27%)	36 (10.4%)	216 (62.6%)
NSAIDs’ long-term use may cause heart disease.	113 (32.8%)	33 (9.6%)	199 (57.7%)
Paracetamol overdose can lead to liver failure.	141 (40.9%)	21 (6.1%)	183 (53%)

**Table 4:** Assessment of general population’s knowledge about the contraindicated conditions with a high level of NSAIDs consumption. NSAID: non-steroidal anti-inflammatory drugs

Case	Yes, they should avoid frequent NSAID self-use.
A person who has poor kidney function	219 (63.5%)
A person who has a gastric ulcer	195 (56.5%)
A person who has hypertension	90 (26.1%)
A person who has a history of ischemic stroke	89 (25.8%)
A person who has bronchial asthma	49 (14.2%)

## Discussion

The findings of this study suggest that OTC use of analgesics is highly prevalent among the population of the eastern province of SA (93%), except for the 7% of the study population who reported never using these drugs without a prescription. The percentage is higher than what was described in four different countries in previous studies [14-17]. The most commonly used medication without a prescription was paracetamol (91%), which is a common finding, but in this study, a higher percentage was reported than the percentage reported in some studies in sub-Saharan Africa and Belgium (68.8%) [18,19]. This high prevalence of NSAID use is most probably due to its wide availability, low cost, and excellent safety profile [18].

Although the prevalence of OTC analgesic use was high, a low frequency of repeated use was reported in this study: 49.3% of the participants rarely used them, and 19.4% used them only every few months. Daily use (6.7%) was lower than what was reported in a study of the Italian population (18%) [20]. In this study, the female gender was associated with a higher frequency of analgesics use (p = 0.017). This was consistent with the findings of three previous studies [19-21]. This is understandable as past research showed that females exhibit a higher sensitivity to pain than males [22]. In addition, menstrual cycle pain was an additional indication for 36.9% of the females in this study. A higher level of education was significantly associated with a lower frequency of analgesics use (p = 0.000), which is in line with multiple previous studies [16-18].

Although the majority had a history of analgesic self-use, only 26.1% of the participants knew more than three side effects. In comparison, even lower percentages of awareness were demonstrated in Urbana, United States, where only 19.8% knew about more than three side effects [14]. The only sociodemographic factor that had a significant relationship with a high level of knowledge was being a city resident (p = 0.030), consistent with a similar study [23]. Better health information access is a likely explanation for this phenomenon [24].

One finding that needs to be highlighted is that 243 participants (70.4%) came to know one or more of these medications through a medical doctor, although they did not show a higher level of knowledge than those who knew these drugs from other sources (p = 0.085). This may imply that physicians tend not to provide their patients with much information about such analgesics’ side effects and contraindications. Since doctors are the most important and trusted source of health information for patients, their role in increasing people’s knowledge and awareness needs to be intensified.

Certain side effects were under-recognized. Only 14.2% knew that bronchial asthma patients need to avoid frequent and uncontrolled NSAID use; this is significant because of the risk of triggering asthma attacks or developing intolerance later in their lives [25]. Furthermore, about a quarter of the participants agreed that patients with hypertension and/or ischemic stroke should be cautious about NSAID overuse. Hence, actions must be taken to increase public knowledge in these aspects, particularly, for the groups who are at a greater risk of developing serious adverse effects and those who have been using these drugs for long periods.

This study had a few limitations. First, the sample size was small and needs to be enlarged to increase its statistical power, specifically, the number of participants with a level of education lower than high school. Second, the survey only used the brand names of the most popular drugs; thus, not all brands were included, and consequently, the prevalence of OTC use of some drugs may have been underestimated. Remarkably, a significantly higher number of participants knew about the possible harmful effects of NSAIDs on kidneys than about other side effects; however, it is uncertain whether the former represents a specific public knowledge regarding NSAIDs side effects or just a part of the general belief that overuse of chemical drugs can cause kidney damage. Finally, future studies are needed to investigate the benefits versus the health burden of NSAID self-medication on the population of the eastern province of SA and its healthcare system.

## Conclusions

In summation, although OTC use of analgesics was widely prevalent among the population of the eastern province of SA, the majority of the population tended not to repeat their use frequently. However, the consumption rates of the female gender and the less educated groups were more than their counterparts. Nevertheless, considering that paracetamol and NSAIDs can be easily procured without a medical prescription or physician consultation, the unrestricted use of these drugs, combined with a lack of knowledge about their side effects and contraindications, may lead to devastating health effects and can increase the burden on the healthcare system. Consequently, the results of this study suggest that more than half of the population need better health education and undertake awareness programs so that they can safely practice self-medication. To maximize patient safety, it is recommended that NSAIDs should be consumed at the lowest effective dosage for the shortest possible time. Patients ingesting NSAIDs who are at increased risk of complications require regular monitoring.
